# Nuclear quantum effects in fullerene–fullerene aggregation in water

**DOI:** 10.3389/fchem.2022.1072665

**Published:** 2022-12-15

**Authors:** Sara Panahian Jand, Zahra Nourbakhsh, Luigi Delle Site

**Affiliations:** Institute of Mathematics, Freie Universität Berlin, Berlin, Germany

**Keywords:** nuclear quantum effects, path integral molecular dynamics, PMF of aggregation of hydrophobic particles, fullerene, adaptive resolution simulation (AdResS) method

## Abstract

We studied the effects of the quantum delocalization in space of the hydrogen atoms of water in the aggregation process of two fullerene molecules. We considered a case using a purely repulsive water–fullerene interaction, as such a situation has shown that water-mediated effects play a key role in the aggregation process. This study becomes feasible, at a reduced computational price, by combining the path integral (PI) molecular dynamics (MD) method with a recently developed open-system MD technique. Specifically, only the mandatory solvation shell of the two fullerene molecules was considered at full quantum resolution, while the rest of the system was represented as a mean-field macroscopic reservoir of particles and energy. Our results showed that the quantum nature of the hydrogen atoms leads to a sizable difference in the curve of the free energy of aggregation; that is, that nuclear quantum effects play a relevant role.

## Introduction

The aggregation of large hydrophobic nanoparticles in water is a subject of interest for its technological and environmental relevance. In particular, the *C*_60_ fullerene, which is produced in a massive manner by, for example, the arc discharge of graphite electrodes (Montellano Lopez et al., 2011), is the most studied hydrophobic nanoparticle in water, both experimentally (Labille et al., 2009; Chae et al., 2010; Ma et al., 2010; Meng et al., 2010; Voronin et al., 2014) and theoretically (Li et al., 2005a; Li et al., 2005b; Maciel et al., 2011; Zangi, 2014; Makarucha et al., 2016). In this context, the potential of mean force as a function of the *C*_60_ fullerene–fullerene distance (PMF), that is, the ensemble-averaged fullerene–fullerene space-dependent force (Kirkwood, 1935; Darve, 2006), has been studied using several classical MD approaches (Makarucha et al., 2016). The PMF explains, in terms of (free) energy cost, the process of aggregation of the fullerene molecules, that is, how the two solutes reach aggregation by breaking the hydrogen bonding network of water and coming near each other. Simulation results based on classical models showed that aggregation eventually occurs without any significant energy barrier. However, the classical models used in previous work do not explicitly describe any quantum feature of water and, thus, cannot account for its potential effects on the strength or flexibility of the hydrogen bonds. In this context, the question of interest is whether the use of a quantum molecular model leads to different results compared to a corresponding classical model in the aggregation process. When a long-range interaction between the carbon atoms of the fullerene and the oxygen atoms of water is used to model the system, water-mediated effects are not relevant in PMF determination (Li et al., 2005b); thus, one can conclude that nuclear quantum effects of water are not likely to play a key role. However, when a purely repulsive C-O interaction is used to model the system, the aggregation process is dominated by the water-mediated effects (Li et al., 2005b); therefore, nuclear quantum effects may become relevant. Experimental results promote the hypothesis that water-mediated effects actually regulate the aggregation (Voronin et al., 2014). The present study tested the relevance of the quantum nature of the hydrogen atoms in the *C*_60_-*C*_60_ aggregation process at room conditions by modeling the C-O interaction as a purely repulsive interaction. This study applied the PIMD technique within the Adaptive Resolution approach (AdResS) (Praprotnik et al., 2005; Praprotnik et al., 2008; Wang et al., 2013; Agarwal et al., 2015; Delle Site and Praprotnik, 2017; Delle Site et al., 2019; Cortes-Huerto et al., 2021). The AdResS technique reduces simulation costs by requiring high (quantum) resolution only in the mandatory solvation region, while the rest of the system is treated at a lower resolution and a small computational cost. The size of the high-resolution region can be automatically and precisely defined by the AdResS method (Lambeth et al., 2010). Our results showed that, at the qualitative level, the PMF calculated with the quantum model did not differ from the PMF calculated with the various classical models; however, a one-to-one quantitative comparison with the TIP4P rigid model; i.e., the closest classical model to our quantum model, showed a sizable difference. Specifically, the depth of the minimum of the PMF curve differed such that one could see the classical model building a strong rigid cage around the aggregated fullerene molecules (deeper minimum), while in the quantum case, the H-bonding network was more flexible and easier to break (less deep minimum). These interesting results add to the methodological message of the paper demonstrating the utility of the open system MD approach to make possible tests of this kind with feasible computational resources. This report is organized as follows: we first provide a brief but essential review of the PIMD idea/technique, followed by the essential description of the AdResS/open system approach and its features. Although this method was previously validated for the quantum water model used here, we further validate the method by studying the solvation of a single fullerene in water and compare the results with simulations of reference. As anticipated, the case of a single fullerene also allowed the precise determination of the minimal solvation region of the two fullerene molecules and, thus, automatically fixed the minimum fullerene–fullerene distance in the PMF calculation. The discussion and conclusions close the paper, while the technical and computational details of the simulations are reported in the Supplementary Appendix.

## The essentials of path integral molecular dynamics

**FIGURE 1:**
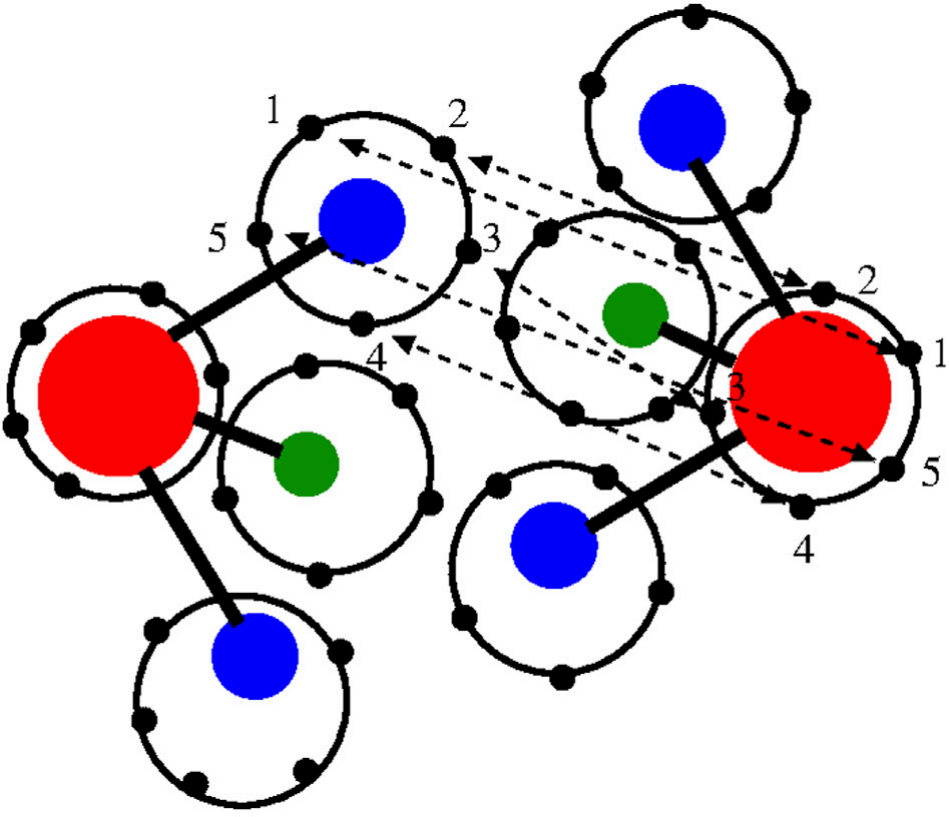
Graphical illustration of the path integral/polymer ring representation of two interacting water molecules of the TIP4P 4-site model used in this work (Habershon et al., 2009). Oxygen (red), hydrogen (blue), and additional site model (green). Each site is represented by a polymer ring; for graphical convenience, only five beads per atom/site are drawn although 30 beads per atom/site are used in the real simulation. Atoms of different molecules interact through bead–bead interactions. The beads involved in the interatomic/intersite interactions are only the beads with the same label (here represented as 1, 2, 3, 4, and 5) of each atom/site. For simplicity, the oxygen–hydrogen interaction is illustrated. The interaction potential has a classical form as the potentials used in the atomistic simulation; however, in this case, the bead–bead interaction is scaled by the number of beads.

Light atoms, such as the hydrogen atoms of water, are strongly characterized by quantum effects that lead to their delocalization in space. The path integral technique is a theoretical tool that satisfactorily describes such effects [see e.g., (Feynman and Hibbs, 1965) and references therein]. In particular, a practical method that approaches realistic systems with satisfactory results is the computational technique known as path integral (PI) molecular dynamics (MD) (Tuckerman, 2010; Tuckerman et al., 2014). In essence, one can use a classical potential and delocalize the interatomic interactions by representing each atom as a polymer ring in which each bead represents an interaction site for the corresponding bead of another atom. The spatial deformation of the ring-polymer during an effectively classical simulation mimics the quantum delocalization of the atom in space (Figure 1); in principle, the larger the number of beads, the more accurate the description of the quantum effect of spatial delocalization.

However, in this representation, each bead counts as a degree of freedom; thus, the cost of simulation, compared to the equivalent classical representation, increases proportionally to the number of beads. This aspect implies a sizable increase in the overall simulation costs compared to classical systems. In general, an atom requires at least 16 beads for a first approximation of a realistic quantum representation. Thus, simulations of a system with 1,000 water molecules represented by a three-site water model with each atom represented by a ring-polymer of 16 beads (thus, 48 degrees of freedom per molecule) become essentially prohibitive, although in practice 30–32 beads are considered the standard for trustworthy simulations (Agarwal and Delle Site, 2015). However, such calculations are expensive and, in particular, for the case of the fullerene–fullerene PMF calculations in the present study, are prohibitive using standard computational resources. Overcoming this challenge requires the use of simulation tools that drastically reduce the mandatory degrees of freedom but provide reliable results. One such method is the recently developed open system MD technique (Delle Site et al., 2019) based on the AdResS technique which has been extensively tested regarding its merging to PIMD (Poma and Delle Site, 2010; Poma and Delle Site, 2011; Potestio and Delle Site, 2012; Agarwal and Delle Site, 2015; Agarwal and Delle Site, 2016; Evangelakis et al., 2021).

## The basics of the adaptive resolution technique

**FIGURE 2:**
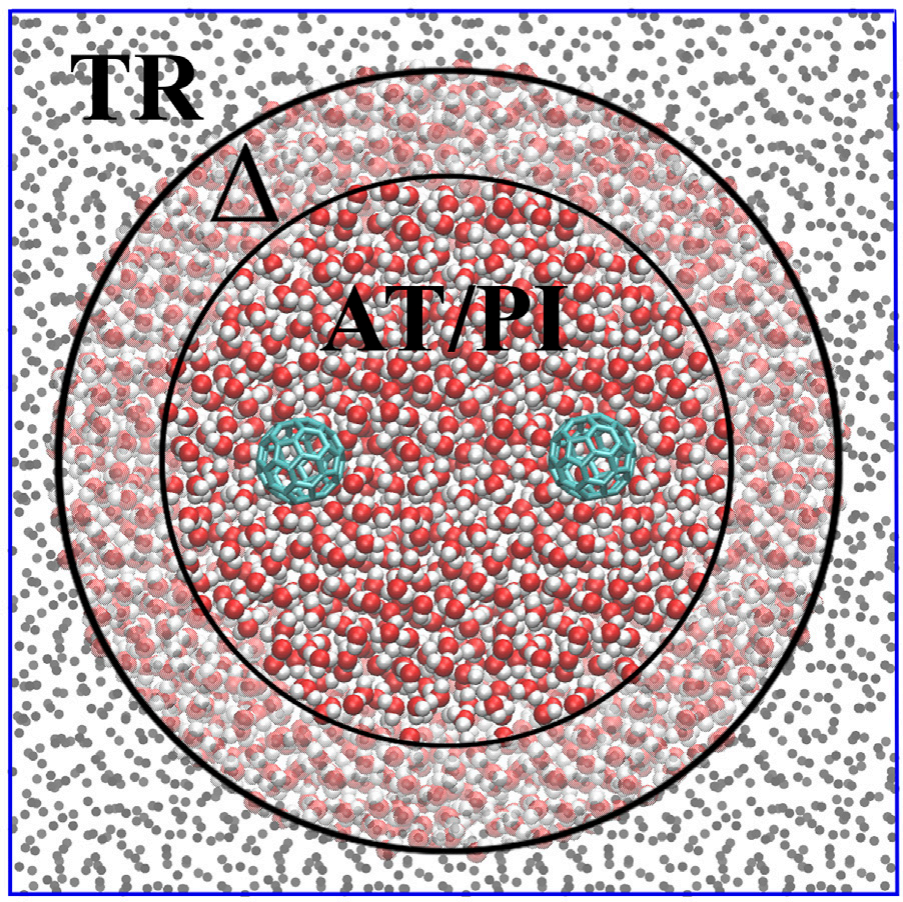
Graphical illustration of the AdResS model for the simulation of liquid water solvating two fullerene molecules.

AdResS treats an open subregion of the simulation domain at full quantum resolution and the rest as a thermodynamic reservoir of energy and particles, that is, as a large domain of non-interacting particles (tracers) thermalized by an external thermostat [the latest version is described in Delle Site et al. (2019) and Evangelakis et al. (2021)]. Figure 2 illustrates the concept, showing a high-resolution region (PI) embedded in a (usually) much larger region of tracers (TR) thermalized by an external reservoir that assures the correct thermodynamic conditions. Between the high-resolution and tracer regions is the so-called Δ (transition) region in which the molecules are at high resolution and experience the external (one-body) thermodynamic force. This force, together with the action of the thermostat, assures the physically consistent exchange of particles between the high-resolution and tracer regions. In essence, the additional force corrects from any difference in the chemical potential between the different regions and ensures the exchange of particles at the chemical potential of a reference (full high-resolution) system. The calculation of the thermodynamic force is performed self-consistently during the equilibration run of the AdResS system (Poblete et al., 2010; Fritsch et al., 2012; Wang et al., 2013; Agarwal et al., 2014; Gholami et al., 2021a; Gholami et al., 2021b). Tracer particles entering the Δ region acquire the chemical structure of the water molecule and the corresponding path integral resolution; on the contrary, molecules leaving the Δ region for the TR region lose their high resolution and become non-interacting particles. Recent results have demonstrated the reliability of this technique for the four-site water model used here with 30 beads per atom, which means that molecules entering the TR region lose 120 degrees of freedom, while molecules entering the Δ region acquire 120 degrees of freedom (Evangelakis et al., 2021). The size of the Δ region is equal to the cut-off distance of the interaction potential such that there is no missing interaction between molecules in the PI and TR regions. The data on the PI region are used to calculate the properties of the open system, while the Δ region represents a sort of artificial region needed to implement the boundary conditions for the PI region so that molecules entering the PI region are automatically equilibrated with the PI environment at the thermodynamic conditions required by the study. The next section considers the solvation of a single fullerene in water and confirmed the reliability of the technique. We also define the maximal region of interest in the fullerene–fullerene aggregation.

## Test of validity of the method: Solvation of a single fullerene in water

**FIGURE 3:**
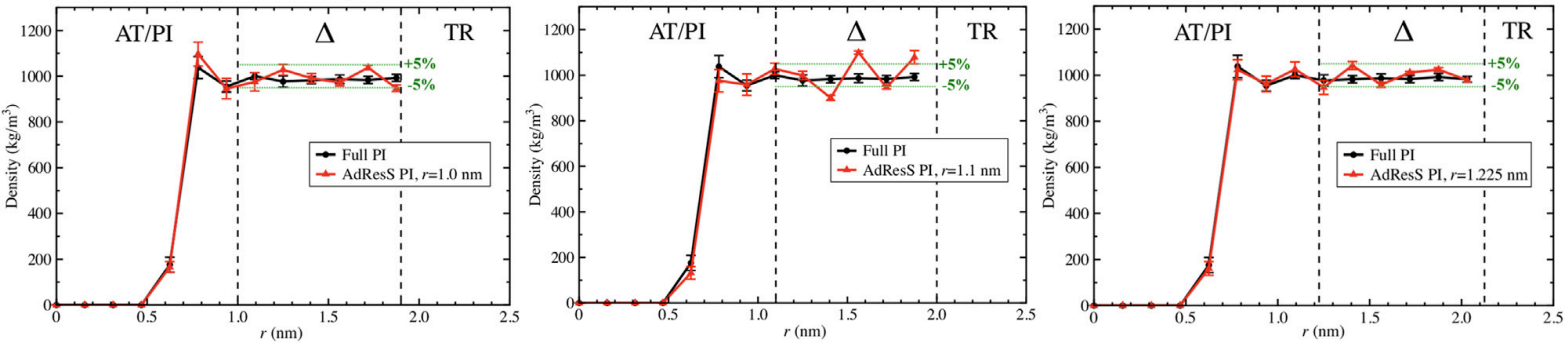
Particle number density calculated in the AdResS setup and compared to the density calculated in the reference simulations for three different radii of the PI region, namely, *r* = 1 *nm*, *r* = 1.1 *nm,* and *r* = 1.22 *nm*. All three figures show sufficient agreement with the reference density. For *r* = 1*nm*, despite a satisfactory agreement in the Δ region, the AdResS density close to the fullerene shows a slight disagreement with the reference density. For *r* = 1.1 *nm* in the Δ region, the accuracy of the density with respect to the density of reference is slightly beyond the 5% threshold. *r* = 1.22 *nm* shows satisfactory agreement over the whole range and the accuracy of the density in the Δ region is within 5% compared to the reference value. 5% is usually considered a satisfactory threshold.

**FIGURE 4:**
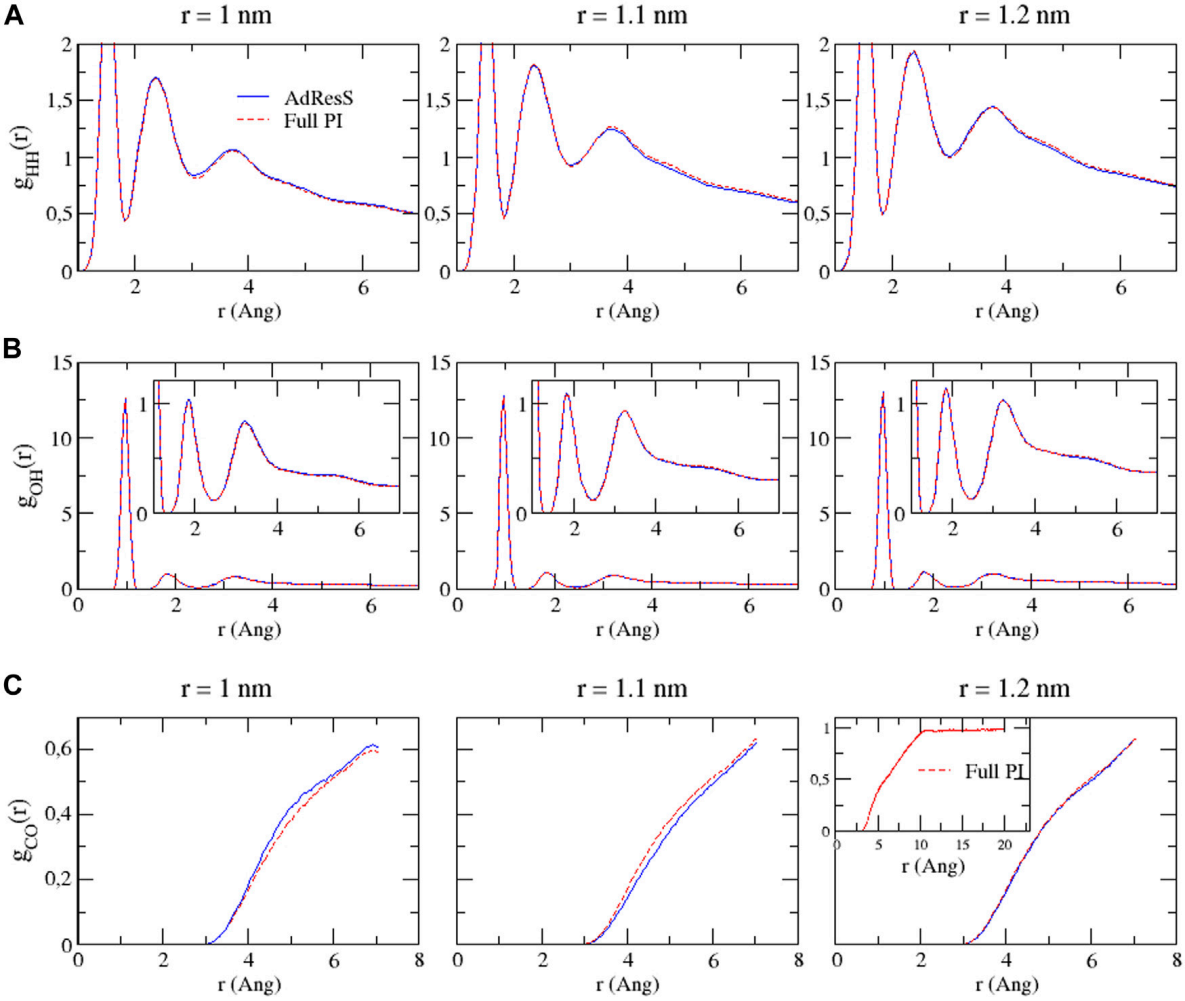
Bead–bead radial distribution functions for hydrogen–hydrogen **(A)**, oxygen–hydrogen **(B)**, and carbon–oxygen **(C)** calculated in the PI region of AdResS and the equivalent subregion of the reference simulation. Since these curves are calculated only in a subregion, they are not normalized.

**FIGURE 5:**
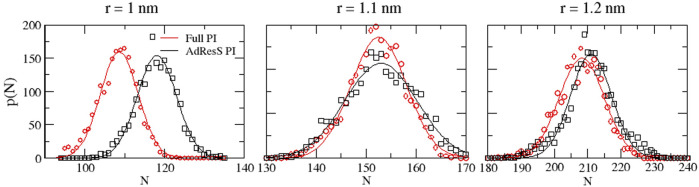
Particle number probability distributions calculated in the PI region and the equivalent subregion of the reference simulation.

To define a physically meaningful open region for the PI resolution region of AdResS, the physical consistency was routinely checked in the AdResS: 1) the water density in the *AT* + *Δ* region should reproduce, within some numerical accuracy, the full reference PI simulation value. The thermodynamic force in Δ ensures that (1) is satisfied. 2) The radial distribution functions should reproduce, within some numerical accuracy, the reference full PI simulation value. These functions represent relevant structural properties that characterize a liquid and its solvation action at certain thermodynamic conditions. In addition, at the statistical mechanics level, their combination expresses the probability distribution function of the system in configuration space up to the two-body approximation (Wang et al., 2013; Agarwal et al., 2015; Evangelakis et al., 2021). 3) The probability distribution function of the particle number in PI, *P*(*N*), must be consistent with *P*(*N*) of an equivalent subregion in the full reference path integral simulation so that the exchange of particles between the PI region and the reservoir (TR) is physically consistent. The concurrent fulfillment of 1, 2, and 3 assures that the explicit quantum degrees of freedom of the PI region are sufficient to reproduce the key features of solvation, while the explicit quantum degrees of freedom outside this region are not relevant for characterizing its physical property and, thus, can be represented by a generic thermodynamic bath. The size of the PI region automatically defines the minimal extension of the mandatory solvation shell and the maximal fullerene–fullerene distance in the PMF calculation (Delle Site, 2022). The maximum fullerene–fullerene distance of interest in a PMF calculation can be accurately determined by the minimum size of the region around each fullerene. Here, water molecules, with their quantum degrees of freedom, directly influence the behavior of the fullerene; beyond this distance, water acts only as a thermodynamic bath and the corresponding hydrogen bonding structure has no direct effect on the fullerene. Regarding the PMF calculation, if the maximum fullerene–fullerene distance is equal to the sum of the radii of the smallest mandatory solvation shells of the single fullerenes, then automatically for larger distances, the two fullerenes do not experience the perturbation of the hydrogen bonding network caused by the other; thus, distances beyond these maximal values are of no interest in the PMF calculation. Figures 3–5 show the calculation of the water density, the various radial distribution functions, and the *P*(*N*) for three different sizes of the PI region. The case of 1.22 *nm* agrees in a highly satisfactory manner with the results of the reference full path integral simulation; thus, it validates the technique as reliable to simulate a physically consistent open region. Moreover, 1.22 *nm* represents the mandatory solvation region and implies that 2.44 *nm* is the largest fullerene–fullerene distance to be considered in the PMF calculation.

## PMF of aggregation of two *C*_60_ molecules

**FIGURE 6:**
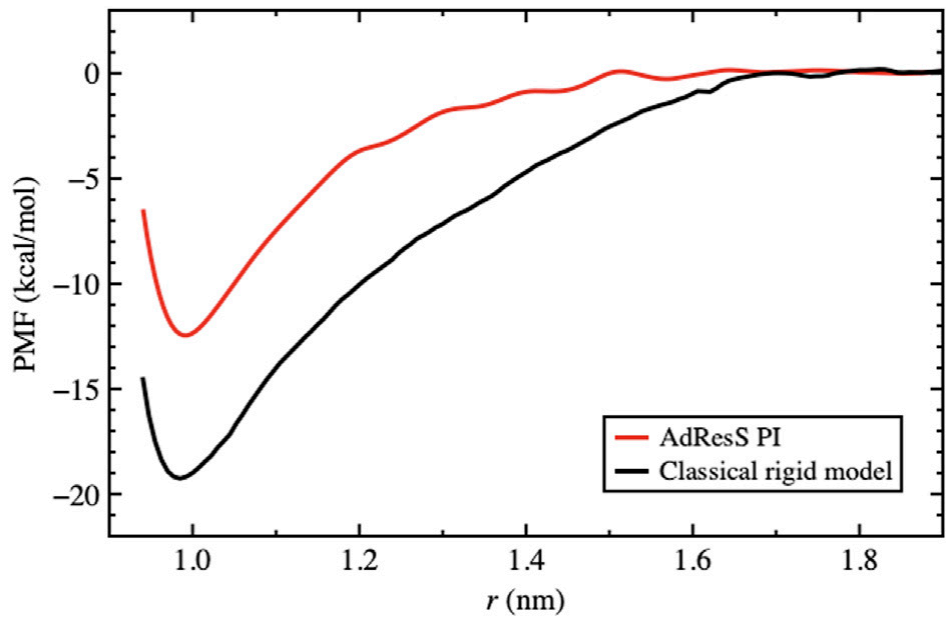
PMF for the path integral model using AdResS compared to the reference full atomistic classical simulation. The PMF is calculated as a function of the distance between the centers of mass of the *C*_60_ molecules. The zero of each curve was chosen to be the corresponding bulk solvation energy, that is, the value of the PMF at the plateau.

**FIGURE 7:**
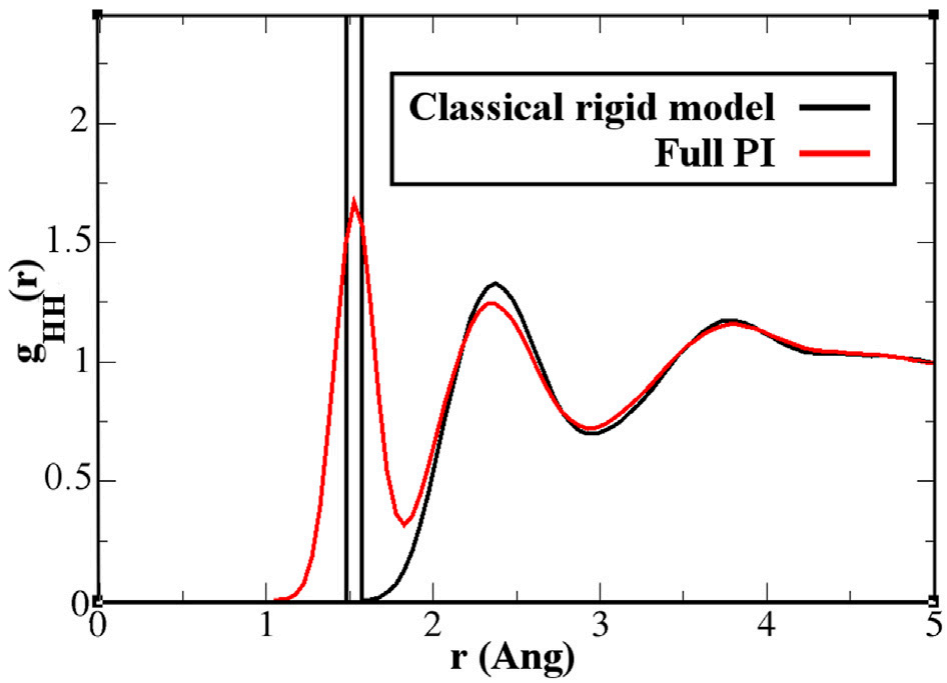
Hydrogen–hydrogen radial distribution function for a pure water system. The classical rigid model (black line) has a first sharply localized peak, while the quantum model (red line) spreads the probability over 1 Å. Further effects are visible, although in a light form, also beyond the intramolecular and first neighbor molecule environment.

As discussed previously, for the solvation of two fullerene molecules, the radius of the mandatory solvation shell is twice that of the single fullerene molecule, that is, 2.44 *nm*. This is also the maximal distance that must be considered for the calculation of the PMF. Figure 6 shows the PMF curve calculated for the quantum model with the PIMD-AdResS simulation, compared to the equivalent classical rigid model. Qualitatively, the aggregation process does not differ in the two cases and the aggregation eventually happens without any significant energy barrier. However, the aggregation in the classical model is energetically more favorable than in the quantum model as the two fullerene molecules approach a closer distance. Once the two fullerene molecules have come in contact, the system falls into a deeper minimum for the classical simulation compared to the quantum case. Thus, the aggregated fullerene molecules are more stable in the classical case compared to that in the quantum case, with a substantial difference in (free) energy of about 7 *kcal*/*mol*. At this point, the quantum model is the direct extension of the classical model, that is, its force field is enhanced by the intra-molecular flexibility (OH bond stretching and HOH angular potential) together with the ring polymer representation of the atoms. The straightforward implication is that the molecular flexibility and the quantum delocalization of the H atoms can sizably influence the (re)organization hydrogen bonding network. For a purely repulsive C-O potential, as used in this study, the aggregation is driven by water-mediated effects; in other words, by the reorganization of the OH-bonding network as the two fullerenes approach each other. The curves in Figure 6 suggest that the degree of reorganization of the OH-bonding network passing from two cages localized around each fullerene, when the fullerenes are far apart, to a large cage that embeds both, once they aggregate, is higher in the classical case than in the quantum case. This idea was also hypothesized previously (Agarwal et al., 2017). Agarwal et al. (2017) also reported a less structured OH-bonding network in the quantum case compared to the classical case. The authors speculated, based on experimental data, that this result may imply a different characterization of aggregated *C*_60_ molecules when quantum effects are considered. In that study, calculations of the aggregation process were not yet possible using standard computational resources and were defined as “feasible in the near future.” The current results fill this gap and provide a quantitative argument for their hypothesis. A detailed analysis of the structure and dynamics of the bonding network would require the calculation of time correlation functions to explain in detail the dynamics of the aggregation. Such a study, which requires much longer trajectories and the careful use of the thermostat only in regions where the dynamics is not investigated, goes beyond the scope of the present study, which aimed to characterize only the static structural properties of aggregation. In this context, the effect of the flexibility of the quantum model becomes evident in the hydrogen–hydrogen radial distribution function (Figure 7). The hydrogen atoms are the true quantum particles of the systems. In their spatial correlation, the quantum delocalization and the induced flexibility of the bonds are clearly expressed. Within the range of $1.0-2.5\overset{{}^{\circ}}{A}$ , the well-structured classical model differs from the quantum model, in which the probability is spread across the whole range. Regarding the technical advantages of the AdResS, the explicit computational gain is still modest compared to its full potential as the parallelization of the code is not yet optimized.

The straightforward comparison with full path integral simulations currently leads to a factor 3. Although not yet optimal, it is already a non-trivial gain as it reduces the requested computational resources to one-third. This difference becomes significant when a large number of calculations are required, as shown in the present case for the determination of the PMF. The additional advantages of this method include the possibility of determining the maximum distance required in a PMF by reducing the need to sample distances that are not relevant but that cannot be excluded *a priori*. Finally, the drastic reduction in the number of degrees of freedom requires a much lower allocation memory, while full path integral simulations would require so much memory that would *a priori* prevent groups without significant computational resources from performing such simulations.

## Conclusion

We applied the open system MD technique based on the AdResS protocol to study the aggregation of two *C*_60_ fullerene molecules in water considering quantum nuclear effects. After validating the simulation techniques and the corresponding technical set-up, we determined the PMF as a function of the centers of the mass distances of the two solutes. These calculations were performed for the quantum case and for the classical case where molecules are modeled as rigid objects. Only purely repulsive interactions between water and the *C*_60_ molecule were considered. In such cases, water-mediated effects have been shown to play a major role. In the case of a potential with an attractive part, this part would play a key role in the aggregation process; thus, the role of the H-bonding network becomes negligible. The difference in the PMF curve of aggregation was qualitatively similar, that is, aggregation occurs without barriers. However, quantitatively, the difference was sizable. This result can be interpreted as the combined effect of the molecular flexibility and the quantum delocalization of H atoms in the reorganization of the H-bonding network in the quantum case. Thus, nuclear quantum effects are very relevant in the aggregation process if a purely repulsive fullerene–water potential is used to model the interaction. From the methodological aspect, the results of this study demonstrated that the open system MD approach can significantly reduce the computational resource requirements, thus permitting studies to be performed that would otherwise be significantly more expensive.

## Data Availability

The raw data supporting the conclusion of this article will be made available by the authors without undue reservation.
