# Supplementary material for: Nuclear quantum effects in fullerene–fullerene aggregation in water
Source: Front Chem. 2022 Dec 15;10:1072665. doi: 10.3389/fchem.2022.1072665 (PMC9799252; doi:10.3389/fchem.2022.1072665)
Supplement: Supplementary file 1 [file Presentation1.pdf]

Appendix to: Nuclear quantum effects in fullerene-fullerene aggregation in water

Sara Panahian Jand,[1] Zahra Nourbakhsh,[1] and Luigi Delle Site[1, a)]

Freie Universität Berlin, Institute of Mathematics, Arnimallee 6, 14195 Berlin, Germany

This Supplementary Material contains the technical details of the simulations discussed in the corresponding paper.

[a)] Electronic mail: luigi.dellesite@fu-berlin.de

I. COMPUTATIONAL DETAILS OF THE SIMULATION

In the current study, we use the PIMD technique, tested in our previous work[1,2] presented in Refs 3–5. PIMD simulations are carried out using AdResS methodology for open systems implemented in GROMACS 5.1 package[6]. For the classical simulations, a rigid 4-site water model (TIP4P/2005)[7] is adopted, while a flexible 4-site water model (q-TIP4P/F)[8] with a ring polymer with 30 beads for each atom is employed for the PIMD simulations. In addition, the fullerenes are simulated using Girifalco parameters[9,10]. The LJ parameters of the carbon atoms are taken as $\sigma = 0.3469$ nm and $\epsilon = 0.27640$ kJ.mol^{-1} (based on Ref. 10), and carbons are considered as uncharged particles. For cross interaction water-fullerene, the Lorentz-Berthelot combination rules is employed and it should be noted that all fullerene-oxygen interactions (in both classical and PIMD simulations) are purely repulsive. As quantum nuclear effects in carbon are negligible at room temperature, we do not apply PI simulations for carbons in our study. Therefore, the thermodynamic force for the AdResS simulations is obtained for a water system using 30 polymer ring beads per atom, as discussed in Ref. 2. To study the electrostatic properties, the reaction-field method with dielectric constant of 80 for water is used. The cut-offs for van der Waals and electrostatic interactions are 0.9 nm which is equal to the thickness of hybrid (Δ) region in AdResS. This is consistent with the minimum length required for proper treatment of the Δ region. For the AdResS simulations, spherical boundaries between different regions are applied.

A. Single fullerene case

The single fullerene system is prepared by placing a fullerene (C_{60}) at the center of a cubic box with the initial side lengths of 5.0 nm and solvating with 3960 water molecules with the approximate density of 1000 kg.m^{-3}. First, the energy minimization by the steepest descent method is performed for a system with P=1 bead (using q-TIP4P/F water model). Then, we carry out 1 ns NPT run (using Parrinello-Rahman barostat with time constant of 200 fs) followed by 1 ns NVT equilibration run (employing velocity rescaling thermostat with time constant of 10 fs) so that the system reaches equilibrium. The obtained final box has the

size of 4.9512 nm at each side. The ring polymer evolution of P=30 beads is applied for the final structure, and 1 ns NVT production run with a Langevin thermostat is performed for each of all simulations with Δ region and full PI simulations (time step = 1 fs, the friction constant=10 fs, and temperature = 300 K). As mentioned before in the main text, to compare with the results of the full PI simulations, three different radii of the spherical AT/PI regions are considered as 1, 1.1, and 1.22 nm.

B. two-fullerene case and PMF calculation

Considering the optimum radius of the AT/PI region (1.22 nm) found for the single fullerene case, we place two fullerenes with the separation distance of 2.44 nm from their center of mass. They are solvated in the simulation box with the side lengths of 7.40 nm with almost 13000 water molecules. The radius of the full path-integral resolution region (AT/PI) considered in the AdResS simulations is 2.44 nm. The Δ region has the aforementioned thickness of 0.9 nm around the AT/PI region. The rest of the simulation box is treated at non-interacting tracer resolution. To have consistency, the simulation box for the classical MD simulations (using rigid water model) is considered with the same initial size and the same number of water molecules.

For both the AdResS and classical simulations, the energy minimization is performed followed by 1 ns NPT run and then 1 ns NVT equilibration run with the same parameters used for the single fullerene case. To extract fullerene-fullerene PMF, umbrella sampling implemented in GROMACS package is performed for the final obtained configuration. Two fullerenes are restrained at different distances (windows) using a harmonic potential. $15 - 20$ windows (depending on the system) along the reaction coordinate r, and 0.8 nm $\leq\ r\ \leq$ 2.4 nm are generated. r is the distance between the centers of mass of two fullerenes. To accurately capture interactions at small COM separations for 0.8 nm $\leq\ r\ \leq$ 1.8 nm, the intervals of 0.05 nm and 0.1 nm are selected, while for larger separations the window spacing is 0.2 nm. Applying the umbrella potential, NVT equilibration simulations are carried out using a stochastic dynamics integrator with a Langevin thermostat for 0.5 ns. The force constant used in the umbrella potential is 5000 kJ.mol^{-1}.nm^{-2}.

Following this phase, production simulations over the reaction coordinate are carried out in the NVT ensemble with a time step of 1 fs for 1 ns in each window. Temperature is coupled with a Langevin thermostat to a bath with T = 300 K, the friction coupling constant is set to 10 fs, and reaction coordinate values are saved every 50 simulation steps. At the end, using umbrella sampling and the weighted histogram analysis method (WHAM)[11], PMF profiles are plotted as a function of r from the final 0.85 ns of the MD simulations.

REFERENCES

[1]A. Agarwal and L. Delle Site, "Path integral molecular dynamics within the grand canonical-like adaptive resolution technique: Simulation of liquid water," J. Chem. Phys. **143**, 094102 (2015).

[2]A. Evangelakis, S. Panahian Jand, and L. Delle Site, "Path integral molecular dynamics of liquid water in a mean-field particle reservoir," ChemystryOpen **11**, e20210026 (2021).

[3]M. E. Tuckerman, B. J. Berne, G. J. Martyna, and M. L. Klein, "Efficient molecular dynamics and hybrid monte carlo algorithms for path integrals," The Journal of Chemical Physics **99**, 2796–2808 (1993).

[4]J. Lobaugh and G. A. Voth, "A quantum model for water: Equilibrium and dynamical properties," The Journal of chemical physics **106**, 2400–2410 (1997).

[5]A. Witt, S. D. Ivanov, M. Shiga, H. Forbert, and D. Marx, "On the applicability of centroid and ring polymer path integral molecular dynamics for vibrational spectroscopy," The Journal of chemical physics **130**, 194510 (2009).

[6]M.J.Abraham, T.Murtola, R.Schulz, S.Pall, J. Smith, B.Hess, and E.Lindahl, "Gromacs: High performance molecular simulations through multi-level parallelism from laptops to supercomputers," SoftwareX **1-2**, 19 – 25 (2015).

[7]J. L. Abascal and C. Vega, "A general purpose model for the condensed phases of water: Tip4p/2005," The Journal of chemical physics **123**, 234505 (2005).

[8]S. Habershon, T. Markland, and D. Manolopoulos, "Competing quantum effects in the dynamics of a flexible water model," J.Chem.Phys. **131**, 024501 (2009).

[9]L. A. Girifalco, "Molecular properties of fullerene in the gas and solid phases," The Journal

of Physical Chemistry **96**, 858–861 (1992).

[10]L. Monticelli, "On atomistic and coarse-grained models for c60 fullerene," Journal of Chemical Theory and Computation **8**, 1370–1378 (2012).

[11]S. Kumar, J. M. Rosenberg, D. Bouzida, R. H. Swendsen, and P. A. Kollman, "The weighted histogram analysis method for free-energy calculations on biomolecules. i. the method," Journal of computational chemistry **13**, 1011–1021 (1992).
